# Evaluation of Adjuvant Effectiveness of Alum-Propranolol Mixture on the Immunogenicity of Excreted/Secreted Antigens of *Toxoplasma gondii* RH Strain

**DOI:** 10.34172/apb.2021.066

**Published:** 2020-07-07

**Authors:** Elyar Meshkini, Arash Aminpour, Khosrow Hazrati Tappeh, Shahram Seyyedi, Meysam Shokri

**Affiliations:** ^1^Department of Parasitology & Mycology, Faculty of Medicine, Urmia University of Medical Sciences, Urmia, Iran.; ^2^Cellular and Molecular Research Center, Urmia University of Medical Sciences, Urmia, Iran; ^3^Department of Immunology, Faculty of Medicine, Urmia University of Medical Sciences, Urmia, Iran.

**Keywords:** Toxoplasma gondii, Propranolol, Alum, Vaccine, Excreted/secreted antigens

## Abstract

***Purpose:*** The introduction of novel adjuvants is an important step in attempts to develop a safe and more efficient vaccine. The present study was performed to determine whether the use of a mixed beta-adrenergic receptor antagonist propranolol (PRP) and aluminum (alum), as an adjuvant, have efficacy for *Toxoplasma gondii* vaccine to induce protective immunity in a mouse model.

***Methods:*** Female BALB/c mice divided into five groups were immunized with excretorys-ecretory antigens (ESA) vaccine, alum-ESA vaccine, PRP-ESA vaccine, and alum-PRP ESA vaccine, as well as with phosphate buffered saline (PBS), as a negative control group. The immune responses were evaluated by lymphocyte proliferation assay for measuring delayedtype hypersensitivity (DTH) response and by cytokine assay for evaluating IFN-γ and IL-5 levels. The survival rate of mice in all groups was assessed during a three-week monitoring period after an intraperitoneal challenge with *T. gondii* tachyzoites.

***Results:*** The results showed that mice immunized with PRP, as an adjuvant, could secret a higher level of IFN-γ, which was significant in comparison to other groups. However, mice vaccinated with alum-precipitated ESA antigen had ability to produce an elevated level of IL-5 compared to other mouse groups (*P* ≤ 0.05). Moreover, alum-PRP co-administration together with ESA vaccine resulted in the longer survival of mice.

***Conclusion:*** The findings of this study revealed that the combination of alum-PRP adjuvants and ESA vaccine of *T. gondii* elicits both humoral and cellular immune responses, which are comparable to either alum or PRP alone.

## Introduction

The intracellular protozoan parasite *Toxoplasma gondii* is the causative agent of toxoplasmosis and infects nearly 30-50% of the world’s human population.^1^ Toxoplasma is virulent for human, as well as all species of mammals and birds, being of great medical and veterinary importance.^2^ Notwithstanding many advances in the prevention of toxoplasma infection over the past decades, the disease remains a public health problem.^3^ A prevalence of 10-30% of toxoplasmosis has been reported in North America, South East Asia, Northern Europe, and Sahelian countries of Africa, and a high prevalence has been found in Latin America and tropical African countries.^4^ *T. gondii* infection in human typically has no clinical symptoms, but it may manifest with mild symptoms. The parasite can also cause severe fatal disease in immunosuppressed individuals, including patients undergoing chemotherapy and transplant recipients or in those with deficiencies in T-cell function such as patients with acquired immune deficiency syndrome (AIDS).^5^

The pathways of host-parasite interactions in human have widely been studied. *T. gondii* is an obligate ubiquitous apicomplexan parasite establishing both the acute and chronic infections.^4^ The acute stage of the infection is caused by the rapidly dividing forms of the parasite called tachyzoites.^6^ After infection, tachyzoites enter a variety of cells and multiply until the disruption of host cell.^7^ Within the infected cells, tachyzoites develop into the membrane-bound intracellular compartments termed the parasitophorous vacuole. These specialized vacuoles prevent fusion with endosomes and lysosomes and subsequently provide a protective barrier against degradation by the host cell, which gives rise to the initiation of growth phase.^8^ The process of invasion ends with the necrotic death of the cell, and parasites release into the extracellular environment. The free tachyzoites can then continue by multiplying anywhere within the nucleated cells and rapidly spread throughout body tissues.^9^ Dubey et al have emphasized that the host control of toxoplasma proliferation renders the cytolytic tachyzoites, to convert them into the slowly replicating bradyzoites.^10^ The parasite in this dormant stage cannot elicit host immune response, and it is associated with chronic infection whereby the parasite continues to growth but at a lower rate than the invasive tachyzoite form. When the immune system is weakened, bradyzoites can convert back to the tachyzoites, leading to the reactivation of a latent infection and later complications such as fatal toxoplasmic encephalitis, myocarditis, and pneumonitis.^11,12^

Yamamoto et al have mentioned that the excretory-secretory antigens (ESA) are likely the key players in the pathogenesis of *T. gondii*and the immune escape of the parasite.^13^ A system of three sets of secretory organelles is actively engaged in the attachment and penetration oftoxoplasma to the host cell.^14^ These organelles have previously been identified as suitable candidates for vaccination against *T. gondii*. The tachyzoite ESA has been proved to stimulate cell-mediated protective immune response much better than the soluble or cyst antigen, and the vaccines harboring ESA antigen have been shown to be simple and induce protection against *T. gondii.*^15^ Under normal circumstances, the acquired immune response driven by the Th1 lymphocytes plays an essential role in reducing the parasite burden.^16^ It has been recognized that interferon gamma (IFN-γ)- and interleukin (IL)-12- producing cells mediate the development of Th1-type immunity.^17^ Macrophages in innate immunity contribute to the control of toxoplasma parasite infection as they are the major sources of IL-12 and stimulate various cell types to produce IFN-γ, the major mediator of resistance to this organism.^18^ The IFN-γ secreted by natural killer (NK) cells and T cells after *T. gondii* infection promotes the differentiation of T cells into the Th1 phenotype and also provides a major signal for the activation of macrophage microbicidal activities.^19^ Meanwhile, IFN-γ has been suggested to control the emergence of free tachyzoites in chronic infections with *T. gondii.*^20^ Given the contributing role of cell-mediated immunity in protection against toxoplasma infection, effective vaccination against *T. gondii* depends mainly on the induction of IFN-γ-driven cellular immune reactions to provide optimal immunological responsiveness upon infection with this particular parasite.

Despite extensive research efforts in vaccine experiments, development of a safe and strong vaccine that protects against *T. gondii* infection is still required. Vaccine development attempts need to consider novel approaches, particularly new vaccines with optimized immunization protocols. One such strategy for improving vaccine potency is the use of appropriate adjuvants to induce long-lasting immune response.^21^ Aluminium salts (referred to as alum) are the only ones licensed for human vaccination formulations by the United States Food and Drug Administration (FDA). Khorshidvand et al have implied that alum has also been served as an adjuvant in human vaccines for more than 70 years.^22^ In spite of the fact that several vaccine adjuvants are more potent than alum, they have not been approved for human use because of their toxicity.^23^ The immunostimulatory features of alum-containing vaccines have initially been shown to link with humoral immunity and Th2 responses; nonetheless, it can elicit cellular immunity via inducing CD8 T-cell proliferation and IFN-γ production.^24^ The properties of alum in the induction of cell-mediated immune response have further been demonstrated in a previous study by Mazloomi et al who evaluated the adjuvant properties of alum alone or in mixture with propranolol (PRP), in order to facilitate the vaccine-induced immune response against the intracellular bacteria *Salmonella typhimurium.*^25^ PRP is a nonselective β-adrenergic receptor-blocking agent and inhibits the functions of norepinephrine and epinephrine.^26^ The sympathetic nervous system is involved in the functional interaction between the neural and the immune system by direct effects of NE and epinephrine on immune cells expressing adrenoreceptors.^27^ Stimulation of β_2_-adrenoreceptors inhibits the production of IL-12, IFN-γ, and tumor necrosis factor-α and may actually lead to the suppression of cellular Th1 responses, thereby promoting Th2 and humoral immunity.^28^ Accordingly, the use of a pharmaceutical β_2_-adrenoreceptor antagonist may shift immune responses toward Th1 immunity, which is necessary for protection against certain conditions.

In the current study, we used an animal model with the aim of developing a *T. gondii* vaccine for human. A good vaccination protocol capable of eliciting both humoral and cell-mediated immune responses might inhibit infection by toxoplasma. Co-administration of various adjuvants can provide a stronger stimulus for immune system, hence conferring better protection against the parasite. In spite of various mechanisms of action, adjuvants do appear to increase immune responses to antigens. The components of the adjuvants employed in our experiment were alum and PRP, which act primarily as a depot carrier for antigen and as an active immunostimulant, respectively. We designed our study to investigate the adjuvant properties of either PRP or alum alone and alum-PRP mixture in T cell response and protection against RH strain *T. gondii in vivo*.. To our knowledge, this is the first report of the immunoprotective value of both alum and PRP adjuvants together with ESA antigens to develop a vaccine against *T. gondii*.

## Materials and Methods

### Mice

Female BALB/c mice aged 6–8 weeks old were used for all experiments. Mice were kept under pathogen-free conditions at the Animal Care Facility of Urmia University of Medical Sciences, Urmia, Iran. All study procedures on mice were carried out in accordance with the Animal Care and Use protocol of Urmia University of Medical Sciences.

### Parasites

Tachyzoites of the RH strain were obtained from the peritoneal exudates of mice inoculated with *T. gondii*. Within three days, the parasites were aspirated from the peritoneal cavity, and tachyzoites were released from tissue cysts by forced extrusion using a syringe and a 27-gauge needle. The peritoneal fluids were centrifuged at 100 ×g at 4°C for 5 min and were washed twice with RPMI-1640 medium containing penicillin and streptomycin to remove cellular debris. The pellet yielded was examined microscopically for the presence of tachyzoites, and the parasite count was determined in a Neubauer chamber.

### ESA preparation

About 2 × 10^9^ of the harvested tachyzoites were washed three times by centrifugation (at 750 ×g at 4°C for 15 minutes) in PBS. Subsequently, the purified tachyzoites were suspended and incubated in RPMI-1640 medium with gentle shaking at 37°C for three hours. The fractions were then centrifuged at 1000 ×g for 10 min, and the supernatants were collected from the tubes and quantified for protein concentration using the Bradford assay. The obtained antigens were stored in suspension at -20°C until use.

### Mice immunization

For immunization, 85 BALB/c mice were randomly divided into five groups of 17 mice each. Group antigen included mice immunized with 20 μg/50 μl of ESA antigen suspended in 100 μL of PBS. The mice in group alum and group PRP received 20 μg/50 μL of ESA in combination with adjuvants alum (alum phosphate gel) or PRP (both from Sigma, Germany), prepared by mixing the ESA and 50 μL of PBS containing alum (50 μL) or PRP (40 ng/50 μL), respectively. In the alum-PRP group, the ESA antigen plus 50 μL of alum and PRP at the concentration of 40 ng/50 μL was administered by the subcutaneous injection. Control mice were given 150 μL of PBS. Three identical immunizations were performed at days 0, 10, and 20 in a total volume of 150 μL at each experiment.

### Lymphocyte proliferation assay

Ten days after the last injection, five mice of each experimental and control group were selected to determine the lymphocyte proliferative responses *in vitro*. The spleens of mice were removed under sterile conditions and homogenized in RPMI-1640 medium to obtain a homogeneous cell suspension. The suspension, centrifuged at 450 ×g for 5 min, was added to ammonium chloride solution 0.9% in 2 ml of RPMI-1640 medium for red blood cell (RBC) lysis, followed by two washings with the aforementioned medium. After centrifugation, the pellet was dissolved in RPMI-1640 supplemented with 10% fetal bovine serum (FBS), and cell viability was measured by Trypan blue exclusion test. About 10^5^ cells/well were seeded onto sterile 96-well culture plates in a volume of 100 μL of RPMI-1640 containing 10% FBS, and the plates were incubated at 37°C in 5% CO_2_ atmosphere. The cells were then stimulated by ESA antigen and added to each well, and the final volume was adjusted to 200 μl per well. After incubation for 48 hours, MTT assay was used to determine the proliferation activity of the cells. Briefly, 20 μL of MTT dye solution (Sigma, Germany) was added per well, and the plates were incubated for an additional four hours. The supernatants were then precisely aspirated, and the crystals were fully solubilized by adding 100 μL of dimethyl sulfoxide (DMSO). The absorbance at each well was measured within 20 minutes at 540 nm.

### Cytokine assay

Ten days after the last immunization, the spleens (n = 25) were removed aseptically from the mice selected from each group, and the spleen cells were homogenized in RPMI-1640 with a homogenizer. Erythrocytes were lysed by treatment with 0.9% ammonium chloride buffer. After centrifugation, the remaining cells were dissolved in RPMI-1640 with 10% FBS. Cells were then counted, and the viability was estimated by Trypan blue test. The spleen cells were dispensed at about 10^5^ cells/well in 0.1 mL of RPMI-1640 with 10% FBS in 96-well microplates and restimulated with antigen (10 μL of ESA + 90 μL of RPMI medium) in culture. Next, 72 hours post incubation at 37°C, the plates were centrifuged at 450 ×g for 10 minutes. Supernatants were collected for measuring IFN-γ and IL-5 and stored at -80°C until use. The concentration of secreted cytokines was estimated by enzyme-linked immunosorbent assay (ELISA) kit (Mabtech, Italy).

### Delayed-type hypersensitivity (DTH) response

Ten days after the last immunization, mice were subcutaneously injected with 100 μL of ESA antigen in left footpad, and PBS was injected into the right footpad of each mouse, as a control. We determined footpad erythema after 24 hours by measuring the thickness of both feet and compared the difference observed in footpads received antigen with those received PBS.

### Challenge of mice and survival rate

Mice from immunized and control groups were challenged by intraperitoneal injection with 2 × 10^3^ live tachyzoites of RH strain on day 10 after the last immunization. The survival rate was then monitored for three weeks.

### Statistical analysis

Data from MTT assay, cytokine level, and DTH response were analyzed using one-way ANOVA, followed by Tukey’s post-test. The survival rate was calculated by Kaplan-Meier method. A *P* value <0.05 was considered statistically significant.

## Results

### Lymphocyte proliferation

**Figure 1:**
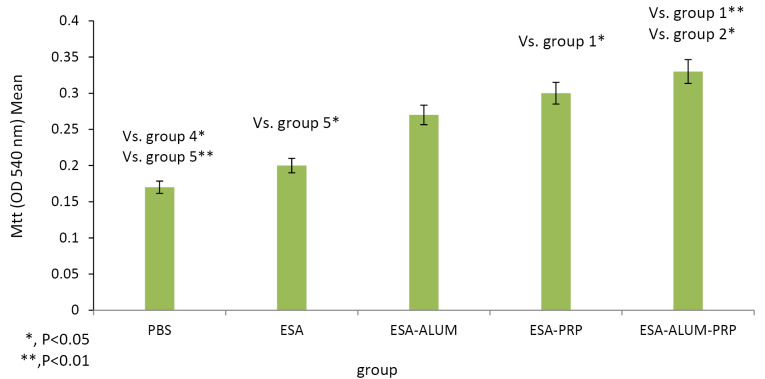
Effect of administering Alum-PRP-ESA mixture on lymphocyte proliferation, Ten days after the final immunization, spleen of individual mice (five per group) were removed and lymphocyte proliferation was evaluated dye the MTT method.

The proliferative responses of spleen lymphocytes in mice treated with ESA formulated with different adjuvants were assessed by MTT assay. Lymphocyte proliferation assays provided an *in vitro* method to evaluate the cell-mediated immunity. Data represented in Figure 1 show that the proliferation of lymphocytes in mice immunized with the ESA vaccine in combination with various adjuvants, including alum, PRP, or alum-PRP, was much higher than the proliferation rate in mice administered with PBS or ESA vaccine alone. Mice immunization with the vaccine combined with alum-PRP mixture or with PRP alone significantly (*P* < 0.01 and *P* < 0.05, respectively) enhanced the proliferative response relative to the control mice that received PBS. The differences were statistically significant between the alum-PRP-immunized and the ESA-administered mice.

### Cytokine levels

**Figure 2:**
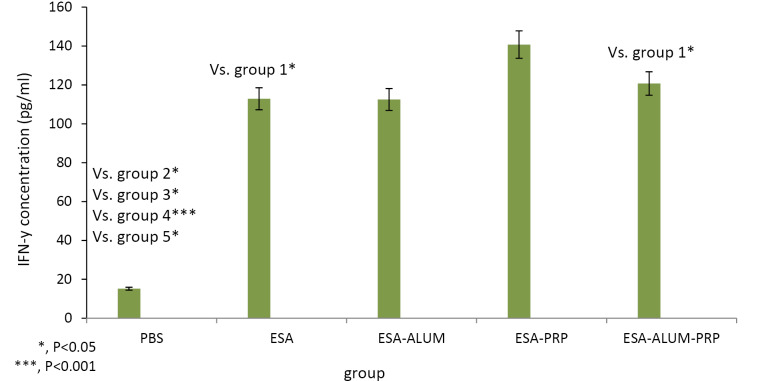
Effect of administering Alum-PRP-ESA mixture on IFN-y production, ten days after the final immunization, spleens of individual mice (five per group)

**Figure 3:**
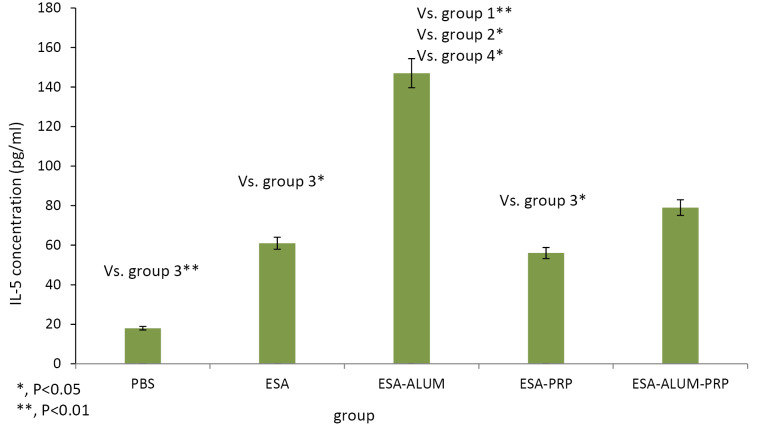
Effect of administering Alum-PRP-ESA mixture on IL-5 production, ten days after the final immunization, spleens of individual mice (five per group)

To detect the cytokine levels, the spleen homogenate samples from the immunized mice were cultured with ESA antigen of tachyzoite RH strain. Concentrations of IFN-γ and IL-5 were measured by ELISA in cell-free supernatants at 72 hours. The results of ELISA test presented in Figure 2 and Figure 3 showed that vaccination of mice with ESA in combination with PRP, as an adjuvant, increased IFN-γ cytokine levels compared to the control and other experimental mouse groups. IFN-γ secreted by splenocytes from mice receiving the vaccines was higher than that of the control mice, but significant differences were found only in PRP mouse group (*P*< 0.001). The difference in IFN-γ amounts between the mice treated with the ESA, ESA-alum, and ESA-alum-PRP vaccines was insignificant. Regarding the results obtained for IL-5, mice injected with the ESA-alum vaccine significantly produced higher levels of IL-5 compared to the IL-5 levels produced in the control, ESA vaccine-received, and PRP groups (*P* < 0.001, <0.05 and <0.05, respectively). However, differences between the ESA-alum-PRP-injected mice and each group were statistically not significant.

### DTH response test

**Figure 4:**
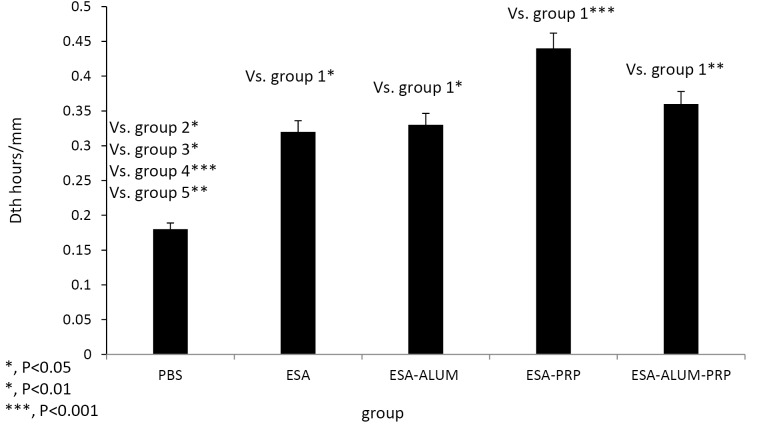
Effect of administering Alum-PRP-ESA mixture on Delayed type hypersensitivity response (DTH) Ten days after the last immunization, mice were injected subcutaneously with 100 μL of ESA antigen in left footpad and phosphate saline was injected into the right footpad of each mouse as a control. (Five per group)

On day 10 after the third immunization, the sensitization of mice to the ESA antigen in different groups was estimated by the footpad assay for DTH response, as described above. The results of footpad assay at 24 hours are shown in Figure 4. Mice showed the highest increase in footpad thickness on immunizations combined with ESA and various adjuvants in comparison to ESA vaccine or PBS alone. Mice in ESA-PRP-vaccinated group developed a higher degree of footpad thickness than other groups, indicating more severe DTH reaction in PRP group.

### Mice immunization and T. gondii challenge experiments

**Figure 5:**
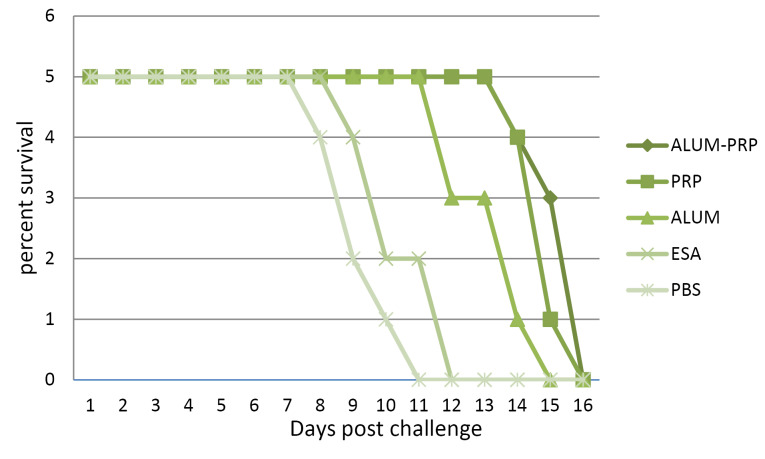
BALB/c mice were infected intraperitoneally with 2000 tachyzoites of the *T. gondii* 10 days after the last immunization. Mortality was monitored daily for a period of 21 days after challenge with strain RH. The percentage of survival in the mice immunized with Alum-PRP-ESA, and challenged with RH strain tachyzoites were significantly higher compared to the mice were immunized with the other groups

Following challenge infection with *T. gondii* tachyzoites, the protective immunity conferred by the various vaccines were determined as the survival rate, which is depicted by a curve in Figure 5. The mortality rate for infection over a period of three weeks was checked daily. Mice treated with alum-PRP or PRP adjuvants had a prolonged survival rate compared to those in the alum, antigen, and control groups. Also, that PBS-administered mice exhibited a minimum survival rate.

## Discussion

Over the past decades, all formulations of vaccines against *T. gondii*were unable to induce protection against and prevent the infection in humans.^29^ Only vaccinations with live, attenuated *T. gondii* parasites appear to be efficacious in reducing tissue cysts after challenge; however, they have been limited to veterinary use, and according to a previous study by Escajadillo et al, such vaccination can develop the breakthrough of infection in human due to side effects.^30^

Our results showed that alum-PRP mixture in combination with the ESA vaccine significantly enhanced the potency of the vaccine. The ESA vaccine when applied with alum-PRP could efficiently stimulate the development of cellular immune responses to the *T. gondii* parasite by increasing IFN-γ and IL-5 production. Previous investigations have also confirmed the relative effectiveness of alum in immunizing animals against *T. gondii.*^31,32^ Studies have suggested that an efficient immunization against naturally occurring toxoplasma infection should be able to skew T-cell activation toward Th1 rather than Th2 pattern.^33,34^ We evaluated the cytokine profile (IFN-γ and IL-5 secretion) to determine Th1/Th2 balance in immunized mice. Mice immunized with alum-PRP adjuvants secreted the large amounts of IFN-γ in response to stimulation with antigen, while a lower level of IL-5 was detected by ELISA assay in the same fractions. The values found were 120 pg/mL for IFN-γ and 70 pg/mL for IL-5 during the 72-h incubation. As already demonstrated, Th1 cells are responsible for cell-mediated immunity, whereas Th2 responses support humoral immunity.^35^ Thus, the immunity induced by the administration of dual adjuvants with the ESA vaccine was biased toward cell-mediated rather than humoral immunity. Furthermore, alum-PRP mixture used as an adjuvant increased the proliferation of lymphocytes and improved resistance characterized by the prolonged survival time of mice challenged with *T. gondii*RH strain.

The specific adjuvant effects of PRP and alum in antigen-specific immune responses were revealed by the administration of either PRP or alum alone in combination with ESA vaccine. Mice immunized with the ESA vaccine with alum were found to secrete more IL-5 (showing the highest level among the groups) than mice receiving PRP in combination with the vaccine. However, using PRP, as an adjuvant, in combination with ESA vaccine could result in significant production of IFN-γ in higher amounts than alum-precipitated ESA in mice. These data were consistent with previous studies ^25^ showing that the stimulatory effect of alum-PRP mixture on the induction of cell-mediated and humoral immunity is considered to be due to the putative functions of PRP and alum, respectively.

Alum salt adjuvants seem to shape the type of acquired immunity to the vaccine antigens into the antibody-mediated protection (humoral type 2 immune response). Conversely, they have relatively little effects on cell-mediated (type 1) arm of the immune responses.^36^ There have been several distinct mechanisms underlying the adjuvant activity of alum *in vivo*. It has already been established that alum has ability to promote Th2 cell polarization, thereby potentiating humoral immune responses.^37,38^ In general, the innate compartment of the immune system is crucial for initiation and progression of adaptive immune responses.^39^ Evidence has reflected that innate cells do release the Th2-skewed cytokine profile such as IL-4-, IL-25-, and IL-6-driving Th2 cell differentiation in response to alum in mice.^40,41^ *In vitro* studies with mouse dendritic cells (DCs) incubated with alum-precipitated ovalbumin have demonstrated that alum directly influences DCs to induce development of Th2 cells from ovalbumin-specific naive T cells. Treatment with anti-IL-1β and anti-IL-18 antibodies blocked Th2 polarization, which is a suggestive evidence that IL-1β and IL-18 may play a role in Th2 response development.^38^ The synthesis and release of these cytokines by innate cells require processing of the inactive intracellular precursors to active form mediated by the enzyme caspase-1 in cytoplasm. It has also been shown that alum activates caspase-1 and promotes the release of mature IL-1β and IL-18.^42^ Furthermore, alum compounds enhance the expression of co-stimulatory (CD40 and CD86) and adhesion molecules (ICAM-1 and LFA-3) on macrophages and DCs, which provide them for antigen presentation and activation of specific T cells.^38^

Owing to the weakness of alum to induce a substantial cell-mediated immune response, which is highly protective against toxoplasma infection, addition of other components to vaccines is required to overcome this pitfall. The combination adjuvants of PRP and alum may generate an optimized immunogen vaccine to achieve the effective type and desired magnitude of immune response to pathogen challenge. Immunization with PRP-ESA in our experiment profoundly increased the IFN-γ level compared to IL-5 levels in this group, indicating that the effects of PRP *in vivo* display a Th1 pattern. The current views concerning the underlying mechanisms responsible for this bias are in part associated with PRP ability to activate the release of proinflammatory Substance P (SP) molecule. SP has been identified as a neuropeptide, the mediator of sensory nerve fibers, involved in neurogenic inflammation.^43^ The proinflammatory capacities of SP generate a specific microenvironment that favors development of Th1 immune responses.^44^ Other mechanism is related to the inhibitory effects on regulatory T cells (Tregs). Tregs, as a subpopulation of T cells, have been described to suppress DC activation.^45^ DCs are critical players in the initiation of immune responses and effector functions of T cells, thereby providing essential help for protection against infectious diseases. The function of DCs in immune regulation depends on their maturation state. Immature DCs in this process can uptake antigens and have capacity to process and present antigen to naïve T cells.^46^ Maturation involves high expression of co-stimulatory molecules and acquisition of the features necessary for T-cell activation, including migration and proinflammatory cytokine production.^47^ Tregs have been displayed to inhibit DCs maturation and function,^48^ which in turn downregulate DCs ability to drive effector T-cell responses.^49,50^ Thus, PRP-induced prevention of Tregs-DC interactions may be another explanation for the enhanced vaccine-induced immune responses.

Survival results indicated that significant resistance against *T. gondii* was conferred by the ESA vaccine when combined with PRP adjuvant or with alum-PRP mixture. Injection of alum alone or ESA vaccine without adjuvant induced less protection, displayed by the lower survival rates in 12.8 and 10.2 days, respectively. Our results suggested that the use of the PRP and alum combined with pathogen-specific antigens very likely provides most effective strategy in directing the immune system to produce the best form of responses to the pathogen.

In 2016, Khorshidvand et al developed a project on Toxoplasma lysate antigens (TLAs) with alum-naloxone and alum-naltrexone adjuvants.^22^ They injected both adjuvants into different groups of BALB/c mice and examined their immunization rate. Ten days after the end of immunization, they measured the cellular immune response and the rate of production of IFN-γ and IL-5. Their results revealed that the increased IFN-γ production improved immunity as well as MTT and DTH assays, and thus the survival rate of immunized mice was higher in the Alum-NLT Group than other groups. In our study, mice receiving alum-PRP together with ESA antigen had higher survival rates than other groups with increased IFN-γ production, indicating a high effect of alum and PRP as adjuvants on mice survival.. In both investigations, antigen-receiving groups with alum produced higher levels of IL-5.

In 2018, Minaei et al investigated a plan for the adjuvant effect of PRP and alum along with total TLAs in immunizing BALB/c mice.^34^ The mice were evaluated for the delayed hypersensitivity test, MTT, challenge, and measurement of IFN-γ, TNF-α, Toxoplasma total IgG, IgG1, and IgG2a. Their results showed that PRP-receiving rats significantly increased IFN-γ, TNF-α, IgG2a, lymphocyte proliferation, delayed hypersensitivity response, and survival. Their conclusion from this design was that PRP, as an adjuvant, together with TLA enhances the cellular immune response. Comparing their study, our own study suggested very high levels of DTH and IFN-γ in mice receiving antigen plus PRP, and based on the MTT assay and the survival rate of the mice, in Minaei’s study, the PRP recipient groups with the ESA antigen responded much better than ours. This discrepancy is probably comes from different antigens used.

In 2019, Abbasi et al. performed a study on *T. gondii* SAG1 antigens in BALB/c mice, and their results indicated that PRP-receiving groups with SAG1 antigen increased IFN-γ production and elevated immune system shift to TH1.^35^ By comparing the MTT assay and the survival rate of the mice, we found that these tests are high in the groups using alum-PRP and higher in the PRP-receiving groups. In Abbasi et al.’s study, IL-5 levels were higher in the TLA-recipient group, whereas in our study, the group used alum with the antigen was the highest. These differences are probably due to our use of alum and different antigens, which alum caused a shift in immunoreactivity and increased IL-5.^35^

## Conclusion

The present study shows that vaccination with ESA antigens of toxoplasma tachyzoites of RH strain in combination with an alum-PRP-mixed adjuvant boosts the level of immunogenicity of the vaccine and importantly can lead to a shift toward Th1 responses associated with strong protection against toxoplasma infection. The adjuvant activity of the mixture of alum-PRP was found to be more potent than the single adjuvant, alum or PRP. Further studies are needed to confirm these results and to determine any benefit in the use of alum-PRP mixture in combination with other pathogen-specific vaccines.

## Ethical Issues

The study was approved by the Ethics Committee of Urmia University of Medical Sciences, Urmia, Iran (approval number: IR.UMSU.REC.1395.14).

## Conflicts of Interest

The authors declare that they have no conflict of interest.

## Funding/Support

This article has been extracted from first author’s MS Thesis of parasitology. This work was financially supported by Urmia University of Medical Sciences as a project (IR.UMSU.REC.1395.14).

## Acknowledgments

The authors appreciate the support of this investigation by the research council of Urmia University of Medical Sciences, Urmia, Iran.
